# Adverse Effects in Patients with Intellectual and Developmental Disabilities Hospitalized at the University Clinical Hospital

**DOI:** 10.3390/jpm12111898

**Published:** 2022-11-13

**Authors:** Montserrat Alonso-Sardón, María Sáez-Lorenzo, Antonio Javier Chamorro, Luz Celia Fernández-Martín, Helena Iglesias-de-Sena, Verónica González-Núñez, José Ángel Santos-Sánchez, Cristina Carbonell, María Fernanda Lorenzo-Gómez, José Antonio Mirón-Canelo

**Affiliations:** 1 Department of Biomedical and Diagnostic Sciences, School of Medicine, University of Salamanca, 37008 Salamanca, Spain; 2 Pharmacological Treatments in Persons with Disabilities, School of Medicine, University of Salamanca, 37008 Salamanca, Spain; 3 Disability and Prevalent Chronic Diseases, Adjunct of Internal Medicine, Faculty of Medicine, University Hospital of Salamanca (SACYL), 37008 Salamanca, Spain; 4 Expert in Social and Communication Skills in Persons with Disabilities, School of Phicology, University of Salamanca, 37008 Salamanca, Spain; 5 Department of Biochemistry and Molecular Biology, Faculty of Medicine, Institute of Biomedical Research of Salamanca (IBSAL), University of Salamanca, 37008 Salamanca, Spain; 6 Instituto de Neurociencias de Castilla y León (INCyL), University of Salamanca, 37008 Salamanca, Spain; 7 Specialist in Traumatology and Radiodiagnosis in Persons with Disabilities, School of Medicine, University of Salamanca, 37008 Salamanca, Spain; 8 Specialist in Infectious Diseases, Salamanca University Hospital, 37007 Salamanca, Spain; 9 Disability and Incontinence, Faculty of Medicine, University of Salamanca Clinical Hospital (SACYL), 37007 Salamanca, Spain; 10 Preventive Medicine and Public Health, Institute of Biomedical Research of Salamanca (IBSAL), 37007 Salamanca, Spain

**Keywords:** care quality, patient safety, adverse effects, persons with intellectual and developmental disabilities

## Abstract

(1) Background: Providing the patient with the health care they need in a personalized and appropriate manner and without adverse effects (AEs) is a part of quality of care and patient safety. The aim of this applied research project was the assessment of AEs as a clinical risk in patients with high social vulnerability such as persons with intellectual and developmental disabilities (PwIDD). (2) Methods: A retrospective epidemiological cohort study was performed on exposed and unexposed groups (the control group) in order to estimate the incidence of AEs in PwIDDs and assess their importance for this category of patients. (3) Results: AEs were observed with a frequency of 30.4% (95% CI) in the PwIDD exposed group, with significant differences to the unexposed group (*p* = 0.009). No differences were observed with regards to gender. Age was as a marker of care risk, with the highest incidence of AEs in the group of 60–69 years. (4) Conclusions: PwIDDs have a high risk of suffering AEs while receiving health care assistance due to their high social and clinical vulnerability. Health care practitioners must therefore be aware of these results and keep these observations in mind in order to carry out personalized, preventive, competent, effective, and safe medical care.

## 1. Introduction

One of the missions of health care systems, regardless of the type of health care system, is the continuous improvement of the quality of care and the promotion of patient safety. Quality of care and patient safety must therefore be firmly linked in any care process.

The turning point in the issue of patient safety at the international level came with the publication of the report “To err is human” by the Institute of Medicine of the United States (IOM) [1,2]. Its conclusions served to raise awareness of the significance and importance of adverse events related to patient health, as well as their economic implications for health care systems. Today, the predominant approach to problems or undesirable events that may occur in health care due to errors, malpractice, and negligence is a reactive one. The first study on the subject is the Harvard Medical Practice Study, developed in New York [3]. The truth is that clinical risk management should revolve around the patient and should identify potential weaknesses and risks in health care, assessing them comprehensively and seeking their root cause. It is therefore essential that any process in health care includes risk management as a basic element for continuous improvement. This makes it possible to plan the organizational environment of health care and its care processes in order to prevent incidents and adverse effects (harm) and, should they occur, to anticipate their impact and thus improve patient confidence and, of course, patient safety. For these reasons, international and national organizations have incorporated patient safety guidelines into their daily practice.

The World Health Organization (WHO), as the greatest exponent on such matters, made the first move in its 55th Assembly held in Geneva in 2002, where they urged the member countries to give the greatest possible attention to the problem and to establish science-based systems that would improve patient safety and quality of care. They also made special emphasis on pharmacovigilance of drugs which most frequently cause AEs. A few years later, the World Alliance for Patient Safety was created, establishing a series of strategies which provided a framework for the adoption of various patient safety measures [4,5]. In the European Union, the Council of Europe presented the Warsaw Declaration on patient safety and the European Health Committee established indications on safety, placing patient safety at the centre of all health care policies [6]. The European Union, through the Luxembourg Declaration, recognizes universal access to high-quality health care as a fundamental human right [7].

Continuous improvement refers to a structured and systematised methodology aimed at achieving a more effective process, reducing the cost of regularly performed activities and improving quality. The end result is observed when changes occur that reduce the incidence of errors and adverse events, improving the organisation’s quality of care and introducing a patient-centred culture that involves all health care professionals [8,9]. In a changing and dynamic scenario such as health care, the organisation must evolve and adapt to new needs. In other words, health care has developed a guarantee of safer and good quality care with the creation of patient safety laboratories [10,11,12].

In Spain, the main national study on adverse events associated with health care was promoted by the Ministry of Health in 2006 and involved 24 hospitals in order to have a representative sample and provide evidence on adverse events in hospitals [13]. The main result indicated that the percentage of adverse events in Spanish National Health System (SNS) hospitals is over 8.4% and that 42.8% of the adverse events observed are avoidable (with 45% being mild and 37.4% being associated with medication). Many health care professionals and preventionists wonder whether adverse effects related to health care could be avoided [14].

People with intellectual disabilities are a heterogeneous group of people who have in common their cognitive-intellectual deficit developing adaptive behaviours to participate and integrate into society and also have specific and frequent health problems according to the disability group. In Spain, when an assessment team establishes a degree of disability of 33%, this is considered a certified and accredited person with disability who consequently has various social rights that give rise to benefits and support that internationally includes the United Nations Convention of 2006 [15].

People with intellectual disabilities remain among the most vulnerable members of society and often face many barriers to accessing health care. They experience significant health problems and risks as a result of social exclusion, discrimination, and isolation [16]. One article provides a human rights-based approach to improving access to quality health care [17]. In a review of articles published in the UK between 2002 and 2010, the authors conclude that PcDIDs have health conditions and impairments that represent health inequalities associated with preventable environmental determinants [18].

Another study carried out in young people suggests that hospital services must adapt to meet their needs, and for this purpose, they have developed an experience called LEARN to provide quality care and not exclude this group of patients from research. [19]. Finally, studies have been carried out on the safety of patients with intellectual disabilities regarding AEs of medicines, and it has been concluded that these people are more likely to have hospitalisations and admissions associated with adverse drug effects [20].

The objective of this study was to determine the incidence of adverse effects in the hospital care of patients with intellectual and developmental disabilities in order to better assess the quality of care they receive in the hospital setting and compare it with the incidence in the general population.

## 2. Materials and Methods

To achieve the stated aim, a study was designed based on the National Study on Adverse Effects (AEs) related to hospitalisation (ENEAS) [13], which was proposed by the Spanish Ministry of Health and was carried out in 24 hospitals of the Spanish National Health System. These hospitals were of varying sizes according to the number of beds: small with under 200 beds; medium from 200 to 499 beds; and large with over 500 beds.

### 2.1. Study Design

The study used a retrospective cohort epidemiological design carried out at the Hospital Clínical University de Salamanca, which had participated in the ENEAS study. The hospital has more than 900 beds with clinical services that include all the legal health specialties existing in Spain.

The IDEA (Identification of Adverse Effects) project’s screening guide was used to identify the AE’s, with a questionnaire drawn up on the basis of previous research and a review of the clinical histories that met exactly 1 of the 19 criteria of the screening guide. These were examined and evaluated by two professionals with a concordance index between the two observers, a physician with specialized training in preventive medicine and public health, and a PhD student in pharmacy from the Department of Biomedical Sciences, with a Kappa coefficient of 0.93. In other words, the concordance between the reviewers was adequate [21].

### 2.2. Target Population and Patients under Study

The study population was PwIDDs who had been hospitalized for more than 24 h, with a medical history in the hospital, and who were subsequently discharged. A representative sample of the PwDID under study was selected, characterized by meeting the requirements outlined by the American Medical Association on Mental Retardation (A.AM.MR.) for PwDID, which states that these individuals have significant limitations in intellectual function and in adaptive behaviors due to lack of practical social and conceptual skills and that they become evident before the age of 18 years [22]. Within PwIDD there are several groups, but the most frequent groups are three: Genetic syndromes, in which the disability manifests itself from birth (the most prevalent and well known are Down syndrome and Rett syndrome in females), whose syndrome is more frequent); people with cerebral palsy who present cognitive deficits and motor disorders with various frequently associated problems, such as epilepsy; and people with autism spectrum disorder (ASD) who have deficits and especially behavioural disorders due to ASD, and a large group of PwIDDs who are undiagnosed. Regardless of their diagnosis, in order to be included they must be accredited as Persons with Intellectual Disability, which in Spain means having a minimum degree of disability of 33%.

### 2.3. Case Definition

In this study, a *case* was defined as any accident recorded in the medical record that had caused or might have caused harm to the patient (PwIDD) and that was related to the nature of the health care and not to the condition or disease for which the patient was hospitalized. An *incident* was considered an event that could have caused harm or complication in other circumstances or that could have promoted the occurrence of an adverse event.

An adverse event (AE) was considered to be any unforeseen and unexpected incident that was recorded in the patient’s medical record and that might have caused injury and/or disability and/or prolongation of hospital stay and/or death as a consequence of the health care and not the patient’s illness. To determine whether an AE occurred, the reviewers scored the evidence on a scale of 6 (1 no evidence or little evidence and 6 virtually certain evidence). A value of ≥ 4 was required to classify an AE as positive. For avoidable adverse effects, the same point scale was used to classify the AE as avoidable (1 equals no evidence or minimal evidence and 6 equals virtually certain evidence). A score ≥ 4 was required to consider it positive.

### 2.4. Selection Criteria and Sample Size

#### 2.4.1. Inclusion Criteria

Patients (age greater than or equal to 18 years) with DID admitted to the Hospital with a stay of more than 24 h, with clinical history and who were subsequently discharged during 2018 were included. PwDIDs were selected with certification of persons with disability that reaches a cognitive level impairment of at least 33% which is what is considered in Spain as a person with certified and accredited disability and, therefore, grants them various Social Rights and Economic Aids established by the Disability Assessment and Guidance Teams of the Social Services of the Autonomous Communities of Spain [23].

#### 2.4.2. Exclusion Criteria

Patients with IDD admitted to the hospital with a stay of less than 24 h or in emergency or short-term observation units were excluded, as well as those whose hospitalization episode was not reflected in the medical record. In addition, DID patients with a degree of disability of less than 33% or whose medical history was not available were also excluded. An estimate was made of the necessary sample size for an expected incidence proportion of adverse events in hospital care, primary care, and outpatient consultations of 9.3% in the general population, using the following formula n: (2² × p × q)/d², where 2² is used since the desired confidence level is 95%., p is the expected ratio, q is equal to 1-p, and d is the precision. In this case, we wish for a precision of 6%. Substituting the terms, we obtain (4 × 0.093 × 0.907)/0.06² = 94 admissions to determine that proportion. A random selection of clinical histories was carried out by generating random numbers in Excel. The sample was described by the following variables: type of disability, date of admission and discharge, sex, admission diagnosis coded according to the International Classification of Diseases ICD-9-CM, diagnosis-related group (DRG), and age. The information was provided by the admission and registration service. The unexposed cohort was created by pairing with another admission without intellectual disability in the following successive order: the same year, ICD-9-CM diagnosis, DRG, age, and sex. If a match could not be found with the same diagnosis or the same DRG, the match was not considered possible.

During the study period, there were 13 admissions of people with ASD (2%), 105 with Down syndrome (15%), 156 with PCI (22%), and 422 (61%) with intellectual disability, for 696 in total. The researchers decided to address 104 histories to compensate for possible losses. Of these, 6 stories did not match the selection criteria, 4 were found, and 2 could not be matched to cases, so in the end, 92 pairs which were consistent in at least DRG and/or admission diagnosis were analysed. Thus, the sample consisted of a total of 184 PwIDDs; of these, 92 were the Exposed Group (EG) and 92 the Unexposed Group (UG). DRG matched in 92.4%, diagnostic group in 90.2%, sex in 75%, and year in 85.9%. Ages were considered to match when the difference within a pair was less than 5 years, which occurred in 66.3% of pairs.

### 2.5. Study Variables

#### 2.5.1. Variables Linked to Care or Assistance

Variables associated with care or assistance include hospitalization service (medical or surgical), type of admission (urgent or programmed), hospital stay in days, extrinsic risk factors (ERF) (urethral catheter, peripheral vein catheter, central catheter, central catheter peripherally inserted, central vein catheter, parenteral feeding, enteral feeding, nasogastric catheter, esophagogastric percutaneous catheter, tracheostomy, mechanically assisted breathing, or immunosuppressive therapy).

#### 2.5.2. Variables Linked to the Illness or Condition

Disease-associated variables included principal diagnosis according to ICD diseases and surgical procedures and ASA risk score classification according to the American Society of Anesthesiologists.

#### 2.5.3. Variables Linked to the PwIDD

The variables studied in the PwIDD included age, sex and intrinsic risk factors (IRF) (coma, renal failure, diabetes, neoplasia, chronic obstructive pulmonary disease -COPD-, immunodeficiency, neutropenia, liver cirrhosis, drug addiction, obesity, malnutrition, pressure ulcers, malformations, heart failure, coronary heart disease and hypertension).

#### 2.5.4. Variables Linked to Impact

Variables associated with the impact of hospitalization included increased length of stay caused by the adverse event (AE), procedures and treatments added as a consequence of the AE, and functional disability.

### 2.6. Measurement Tools

The forms used for the study of AD were based on the ENEAS Study Guide carried out in Spain in 2005 for AD screening [13] which arose as an adaptation of the Harvard study [3] and other subsequent international studies conducted in Australia, London, Denmark, New Zealand, and Canada [24,25,26,27,28,29,30].

Disability is generally assessed by an official body of the Spanish Institute for the Elderly and Social Services (IMSERSO) of the Ministry of Health and is evaluated in degrees with the percentages of disability by a team composed of a social worker, a clinical psychologist, and a rehabilitation physician who performs the evaluation, assessment, and orientation, and who are collectively called the EVO team [23]. Dependency is assessed with the Barthel Index, which is established by assessing and scoring all the Basic Activities of Daily Living (BADL) such as eating, washing, dressing, toileting, defecation, and urination, transfers, walking on a flat surface, and going up and down stairs. Depending on the score and the level of support needed, mild, moderate, severe, or total dependency is awarded. This is also implemented by the EVO teams, who perform an evaluation and orientation.

Once the pairs were established, the dossiers were requested physically and were systematically reviewed with the clinical history summary form IDEA PROJECT Identification of Adverse Effects and checked for compliance with the 16 intrinsic risk factors (IRF) and 14 extrinsic risk factors (ERF) used by the ENEAS study.

### 2.7. Data Analysis

A description was made of the sample (patients included, excluded, and lost) and of the variables studied. An analysis was carried out on the results recorded by the forms and on the consequences of the AE. Statistical analysis consisted of a univariate analysis for the description of the sample (frequencies for categorical variables; mean, median, standard deviation, and interquartile range for continuous variables) and a bivariate analysis to establish the relationships between the variables (Chi-square to compare proportions; Student’s T-test and analysis of variance/ANOVA to compare means between two or more groups). The prediction model was created by binary logistic regression with the forward method, the appearance of AEs as the dependent variable, and those variables which were found to be statistically significant during the bivariate analysis being independent or explanatory variables. To compare the incidence of AEs among groups, relative risk (RR) and the corresponding Confidence Interval (95%CI) were calculated, giving the range of values for the population of a given variable. Significance of less than 0.05 was used during hypothesis testing. Statistical work was performed with the statistical program SPSS, version 26.0.

### 2.8. Ethical Considerations

This study was performed in accordance with the recommendations given in the Declaration of Helsinki. All study participants were required to maintain confidentiality of the information obtained. Data presentation was aggregated such that in no case was it possible to identify a patient. The study was submitted for consideration to the Ethics and Clinical Research Committee Ethics of the Salamanca’s University Hospital.

## 3. Results

Table 1 shows the sample characterization for the cohort of the EG and UG groups. The mean age was similar in both groups: 50 years in EG and 53 years in NG. 

**Figure 1:**
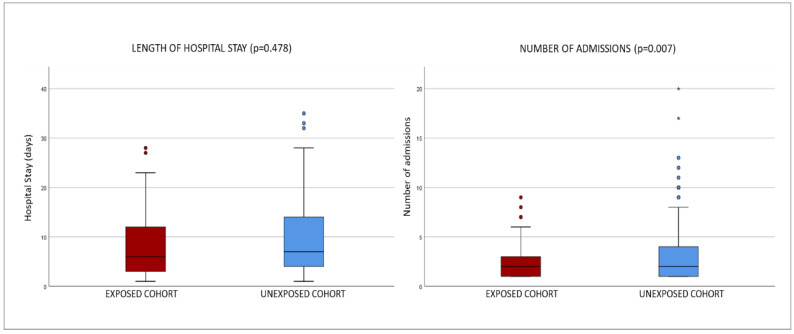
Box plots of hospital stays in both groups: exposed cohort vs. unexposed cohort.

Gender distribution was very similar: 49 men and 43 women in EG, 48 men and 44 women in UG. Average stay was 16 days in EG and 12.5 in UG. Average number of admissions was 2.4 (minimum value-maximum value, 1-9) in EG and 3.5 (minimum value-maximum value, 1-20) in UG. See Figure 1.

Within the patients with disabilities, or Exposed Group (EG*)*, four basic groups exist, all PwIDDs. The largest group was “persons without a specific diagnosis”, with 48 individuals (52.2%); 24 patients (26.1%) were diagnosed as PwIDDs; 16 patients (17.4%) had a diagnosis of Down syndrome; 4 patients (4.3%) had a diagnosis of Autism spectrum disorder.

Most patients had a degree of disability above 70% (143, 77.7%) and above 80% (41, 22.3%), with small differences between the EG and UG. Degree of dependence was as follows: Grade I patients (23, 12.5%), who require little support and that only in specific circumstances; Grade II (142; 77.2%), who have mild and moderate dependency due to needs of daily support for several BADL, several times a day; and Grade III (19, 10.3%), people with severe and total dependency who need help and support for most BADL, often every day.

Significant differences were found in intrinsic risk factors between the cohort of the exposed group (CEG) and the cohort of the unexposed group (CUG), along with high OR/RR in coma, renal failure, diabetes, COPD, neutropenia, hypoalbuminemia, pressure ulcers, and malformations, with the latter having a strong association (OR = 2.1). See Table 2 for intrinsic risk factors (IRFs) and extrinsic risk factors (ERFs).

The predictive model (logistic regression) for intrinsic risk factors associated the dependent variable “onset of AEs” with the following independent or explanatory variables: kidney failure (Exp(B) = 3.134; 95% CI, 1.147–8.565; *p* = 0.026), neoplasm (Exp(B) = 6.805; 95% CI, 2.068–22.392; *p* = 0.002), and hypoalbuminemia (Exp(B) = 4.038; 95% CI, 1.611–10.118; *p* = 0.003). No significant predictive model was found for extrinsic risk factors (*p* > 0.050).

The most notable differences in extrinsic risk factors were urinary catheter, parenteral nutrition, and prior institutionalization, the latter being the strongest (OR = 13.6). This shows that the EG cohort had a higher frequency of admissions with adverse effects during prehospitalisation due to their vulnerability, as they are patients with disabilities with various degrees of dependency, fundamentally grades II and III.

**Figure 2:**
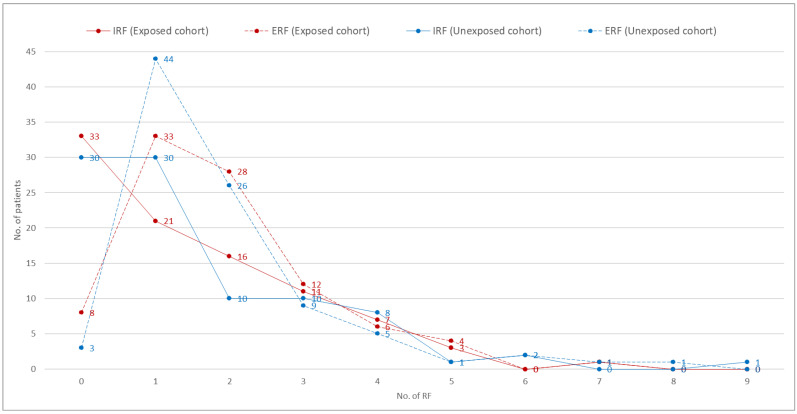
Count of Extrinsic RF (ERF) and Intrinsic RF (IRF): exposed cohort vs. unexposed cohort.

It can be seen in Figure 2 that there was no significant difference between the groups for the T-test, neither for the sum of all IRFs (p = 0.928; mean difference = 0.022) nor for the sum of all ERFs (p = 0.958; mean difference = 0.011).

**Figure 3:**
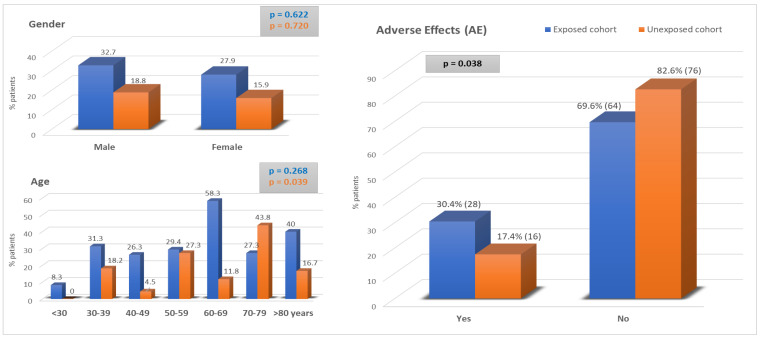
Adverse effects: Exposed Cohort vs. Unexposed cohort.

Finally, as can be seen in Figure 3, the incidence of AEs in the exposed group was significantly higher at 30.4% vs. 17.4% in the unexposed group (*p* = 0.038). That is to say, almost one out of every three patients with intellectual disability presented adverse effects as a consequence of health care, with no significant differences between gender (*p* = 0.954) or age groups (*p* = 0.282), although it is noteworthy that the percentage of AEs was higher in the exposed cohort across all age groups, except for patients between 70 and 79 years of age.

## 4. Discussion

Firstly, the authors are aware that providing the health care required by each patient at a given time and in an appropriate manner is an essential objective of clinical activity, which is often complex because it involves risks, with, at present, the high prevalence of chronic patients with comorbidity and an average of 2.8 chronic diseases in patients over 65 years of age. In addition, there is great pressure on Spanish hospitals and, therefore, this clinical investigation has been carried out in the care context of a hospital.

Secondly, the available data on the overuse of hospital clinical care due to the chronicity and aging of patients oblige us to reflect the inefficiency generated by a saturated health system that consequently conditions the appearance of more adverse effects (AEs). For all these reasons, a broad consensus has been developed to promote quality of care and patient safety [31,32].

Thirdly, we would like to comment that this research work on AE’s is part of studies whose mission is to improve the Quality of Care and Patient Safety, which is a worldwide Public Health problem. This work has tried to estimate AE’s in a population group with high socio-health vulnerability due to the fundamental characteristics of PwDID, such as their intelligence deficit to adapt to the current complex and competitive social life, so they have daily support needs to lead a life as normalized and integrated as possible. On the other hand, the culture of Patient Safety must be approached by abandoning the concept of blame of healthcare professionals, since AE’s are only rarely due to an occasional mistake by professionals. On the contrary, AE’s are usually the result of a set of conditioning factors and causes that are usually avoidable when analysing the complexity of the care processes carried out daily in hospitals. The incidence of AEs observed in the PwDID cohort was 30.4% (95% CI 30.4 ± 5). This incidence rate is much higher than that observed in the 2005 Spanish study of adverse effects in patients from the general Spanish population, which was 8.4% (95% CI: 7.7–9.1), and is also higher than that of other international studies [25,26,27,28,29,30]. These observations can be explained by the characteristics of the people studied, who, as PwDID, have high health and social vulnerability as determined by their genetic, cognitive, and social characteristics that do not allow them to communicate adequately with their environment, much less with an unfamiliar environment such as the health care environment. To this specific intrinsic intellectual vulnerability must be added the extrinsic vulnerability in the family environment with parents who are usually very old as well as the hospital environment which is unfamiliar and complex. All this poses a threat to their safety. We must also add a series of latent care characteristics in the organisation of hospital care and the quality of the available resources that form part of the culture of safety as well as the professionals and teams that carry out their activity in a context of high care pressure due to high demand, which together with the inexperience in this type of patient and the deficit in social and communication skills with this type of patient, can lead to a greater risk of AEs.

The mean age of the IDPs in this study was 50 to 55 years, with a standard deviation of 17.74 years. In the Spanish ENEAs study, the risk of developing adverse effects was greater in patients over 65 years of age, to the extent that its frequency was double that of those under 65 years of age. In this study, as in ENEAS, no gender differences were observed between men and women.

Of the intrinsic and extrinsic factors, malformations stood out with an RR of 2.1. In other words, their genetic idiosyncrasies double the chances of adverse events (AEs) and contribute to a high probability of AEs with this type of patient. This should be taken into account at the start of healthcare and during their hospital stay. AE’s can be avoided by appropriate follow-up, as a large proportion of care-related AE’s are avoidable, with 42.8% being reported as avoidable according to the most important Spanish study [13].

The presence of other comorbidities that facilitate the occurrence of adverse reactions and are not adequately recorded in the ED report should also be ruled out [33,34]. In addition, these patients are often polymedicated and do not always follow a correct prescription with a high prevalence of use of drugs acting on the central nervous system for the treatment of a wide variety of neurological and psychiatric diseases [35,36]. A cross-sectional study conducted in Ireland on a sample of 677 older people with intellectual disabilities over 40 years of age establishes a high prevalence of gastrointestinal pathology and the authors draw attention to the prevalent use of proton pump inhibitors (PPIs) with adverse effects leading to additional comorbidities and the need for regular follow-up and review [37]. Another study associates PPIs with depression [38] while other research in older people reports a high body mass percentage to be significantly associated with poor health-related quality of life and depression, underlining the clinical importance of analysing body mass index in vulnerable older people [38,39]. Another comorbidity that has been evidenced in PcDIDs with Down syndrome is the prevalence of osteoporosis because bone mineral density declines more rapidly in these patients than in the general population [40].

The limitations of this study are several, and the authors are aware of some of them. First, the association between patient severity and AE has not been analysed; however, by analysing age (as a risk marker), hospital stay, and intrinsic and extrinsic factors, an indirect approximation can be made to clinical and care severity [13,41]. Secondly, only PwDIDs with a degree of disability ≥33% were considered because the higher degree of disability may determine a higher risk of AD and justify its high incidence [13,27]. Thirdly, this study was not specifically designed for the observation of AEs due to medication, but it has proven to be effective in identifying this type of AE, which has been one of the most frequently found in various studies to date [33,34,35,36].

Finally, another of the limitation of this research is that epidemiology is not sufficient to explain this phenomenon since it provides the incidence and its distribution, which is just the tip of the iceberg; however, it does not take into account the shortcomings of the health care organisation and its functioning in terms of quality of care and patient safety, which remains submerged, as there are multiple human and organisational factors whose knowledge is difficult to establish and assess [41]. Furthermore, the authors are aware that it is difficult to generalise the results of this study, as this research has considered PwDIDs without taking into account the specific characteristics of patients in the different groups of PwDID associated with specific pathologies and health problems. These should be considered in future research.

## 5. Conclusions

It can be confirmed that patients who are persons with intellectual disabilities should have personalised, preventive, predictive, and participatory care with continuous monitoring when they are hospitalised in order to prevent avoidable AEs in their hospital care. Priority should be given to raising awareness among health care professionals working in the clinical neurology, mental health, and emergency departments, as they are the ones who most frequently attend to these patients.

## Figures and Tables

**Table 1:** Description of the sample.

		Exposed Cohort	Unexposed Cohort	
Qualitative Variables		N (%)	N (%)	*p*-Value ^1^
Gender	Male	49 (53.3)	48 (52.2)	0.883
	Female	43 (46.7)	44 (47.8)	
Service	Medical	64 (69.6)	59 (64.1)	0.709
	Surgical	26 (28.3)	30 (32.6)	
	ICU	2 (2.2)	3 (3.3)	
Disability	Intellectual Disability	48 (52.2)	-	-
	ICP	24 (26.1)		
	Down Syndrome	16 (17.4)		
	Autism	4 (4.3)		
Percentage of Disability	≥70% disability	71 (77.2)	72 (78.3)	0.883
	≥80% disability	21 (22.8)	20 (21.7)	
Disability Grading	Grade I	10 (10.9)	13 (14.1)	0.790
	Grade II	72 (78.3)	70 (76.1)	
	Grade III	10 (10.9)	9 (9.8)	
Quantitative Variables		Mean (±SD)	Mean (±SD)	*p*-Value ^2^
Age		50.5 (±17.7)	53.6 (±18.1)	0.252
Length of Stay		16.0 (±40.9)	12.5 (±21.3)	0.478
Admissions Count		2.4 (±1.8)	3.5 (±3.6)	0.007

^1^ Pearson’s Chi-squared test. ^2^ ANOVA. Statistical significance (*p*-value < 0.05).

**Table 2:** Risk factors: positive responses (Yes).

Intrinsic RF					Extrinsic RF				
	Exp. Coh. N (%)	Unexp. Coh. N (%)	*p*-Value *	RR (95% CI)		Exp. Coh n (%)	Unexp. Coh. N (%)	*p*-Value *	RR (95% CI)
Coma	18 (19.6)	14 (15.2)	0.437	1.155 (0.817–1.634)	Open urinary catheter	-	-	-	-
Renal failure	17 (18.5)	11 (12.0)	0.218	1.263 (0.899–1.774)	Closed urinary catheter	32 (34.8)	27 (29.3)	0.430	1.130 (0.840–1.521)
Diabetes	18 (19.6)	14 (15.2)	0.437	1.155 (0.817–1.634)	Peripheral venous catheter	76 (82.6)	76 (82.6)	>0.999	1.000 (0.683–1.464)
Neoplasia	4 (4.3)	13 (14.1)	0.022 *	0.447 (0.187–0.882)	Arterial catheter	4 (4.3)	4 (4.3)	>0.999	1.000 (0.492–2.031)
Immunodeficiency	2 (2.2)	8 (8.7)	0.051	0.387 (0.111–1.347)	Peripherally inserted central catheter	4 (4.3)	6 (6.5)	0.515	0.791 (0.365–1.714)
COPD	9 (9.8)	8 (8.7)	0.799	1.065 (0.663–1.710)	Central venous catheter	6 (6.5)	7 (7.6)	0.774	0.918 (0.501–1.682)
Neutropenia	3 (3.3)	2 (2.2)	0.650	1.207 (0.581–2.506)	Umbilical catheter (vein)	-	-	-	-
Hepatic cirrhosis	3 (3.3)	4 (4.3)	0.700	0.852 (0.358–2.030)	Umbilical catheter (artery)	-	-	-	-
Drug addiction	-	7 (7.6)	0.007 *	-	Parenteral nutrition	4 (4.3)	3 (3.3)	0.700	1.149 (0.595–2.220)
Obesity	5 (5.4)	5 (5.4)	>0.999	1.000 (0.529–1.891)	Enteral nutrition	-	2 (2.2)	0.155	-
Hypoalbuminemia	19 (20.7)	14 (15.2)	0.337	1.191 (0.851–1.667)	Nasogastric tuve	7 (7.6)	7 (7.6)	>0.999	1.000 (0.580–1.725)
Pressure ulcer	3 (3.3)	2 (2.2)	0.650	1.207 (0.581–2.506)	Tracheostomy	-	-	-	-
Malformations	6 (6.5)	3 (3.3)	0.305	1.357 (0.834–2.205)	Mechanic ventilation	3 (3.3)	5 (5.4)	0.470	0.742 (0.300–1.836)
Heart failure	5 (5.4)	6 (6.5)	0.756	0.904 (0.465–1.756)	Immunosuppressive therapy	1 (1.1)	2 (2.2)	0.560	0.663 (0.133–3.306)
Coronary heart disease	6 (6.5)	6 (6.5)	>0.999	1.000 (0.557–1.795)	Prior institutionalisation	29 (31.5)	3 (3.3)	<0.001 *	2.187 (1.756–2.723)
Hypertension	18 (19.6)	21 (22.8)	0.588	0.904 (0.622–1.315)	Substance abuse	10 (19.9)	33 (35.9)	<0.001 *	0.400 (0.228–0.701)
Sum of IRF (mean ± SE)	1.5 (±1.5)	1.5 (±1.7)	0.928		Sum of ERF (mean ± SE)	1.9 (±1.3)	1.9 (±1.4)	0.958	

* Pearson’s Chi-squared test, statistical significance (*p*-value < 0.05).

## Data Availability

The data used for this study are from the Clinical Histories of the Central Registry of Clinical Histories of the Hospital Clinic Universitario de Salamanca (Spain).
